# Correction to “*Gentiana macrophylla* flavonoids from Tibetan medicine decreases circ_0059665 to alleviate the progression of non‐small cell lung cancer under hypoxia”

**DOI:** 10.1111/1759-7714.15441

**Published:** 2024-09-01

Mi M, Ciren D, Feng M, Zhen L, Jin G. *Gentiana macrophylla* flavonoids from Tibetan medicine decreases circ_0059665 to alleviate the progression of non‐small cell lung cancer under hypoxia. *Thorac Cancer*. 2024;15(1):77–88.

In the Results and Discussion section, the captions for Figures 4 and 7, and references 31 and 32, “miR‐512‐3p” was incorrect and should be corrected to “miR‐512‐5p”.

Circ_0059665 targets miR‐512‐5p, and NOVA2 is a target of miR‐512‐5p heading under the Results section should be corrected as follows:

Subsequently, the underlying targets of circ_0059665 were explored. According to the prediction of Circinteractome and CircBank databases, two miRNAs (miR‐197‐3p and miR‐512‐5p) were identified in the overlapping of these two databases (Figure 4a). After overexpressing circ_0059665, we found only miR‐512‐5p expression was decreased in A549 cells (Figure 4b). The binding sites of miR‐512‐5p on circ_0059665 are listed in Figure 4c. The transfection efficiency of miR‐512‐5p was validated by qRT‐PCR (Figure 4d). Then dual‐luciferase reporter assay showed that miR‐512‐5p mimic notably reduced the luciferase activity of WT‐circ_0059665 vector in H1299 and A549 cells (Figure 4e,f), indicating the interaction of miR‐512‐5p and circ_0059665. The levels of miR‐512‐5p were decreased in NSCLC tissues (Figure 4g), which was negatively correlated with circ_0059665 expression (Figure 4h). Also, its expression was also decreased in H1299 and A549 cells (Figure 4i). Moreover, miR‐512‐5p expression was downregulated by hypoxia and upregulated in response to GF treatment (Figure 4j).

In addition, the TargetScan database predicted that miR‐512‐5p has binding sites on NOVA2 (Figure 4k). Then dual‐luciferase reporter assay also verified the binding between miR‐512‐5p and NOVA2 (Figure 4l,m). NOVA2 expression was higher in NSCLC tissues (Figure 4n), and was negatively correlated with miR‐512‐5p (Figure 4o).

NSCLC cell lines also showed an increase of NOVA2 expression (Figure 4p). Furthermore, hypoxia treatment increased NOVA2 expression in H1299 and A549 cells, which was reduced by GF treatment (Figure 4q).

GF treatment suppresses the tumor growth of NSCLC in vivo via circ_0059665 heading under the Results section should be corrected as follows:

The action of GF in tumor growth in vivo was then studied. As shown in Figure 7a,b, circ_0059665 silencing or GF treatment suppressed tumor growth in mice. In addition, compared with GF treatment alone, the xenograft tumors in sh‐circ_0059665 + GF group were much smaller and lighter. The levels of circ_0059665 and NOVA2 were downregulated while miR‐512‐5p was increased in xenografts after circ_0059665 knockdown or GF treatment; moreover, circ_0059665 knockdown enhanced GF‐induced decreases of circ_0059665 and NOVA2, as well as increase of miR‐512‐5p (Figure 7c–e). In addition, IHC analysis showed the protein levels of NOVA2, c‐Myc, and MMP2 were decreased by circ_0059665 knockdown or GF treatment, and the decrease caused by GF was boosted by circ_0059665 silencing (Figure 7f). Thus, GF treatment suppressed NSCLC growth in vivo via circ_0059665.

In addition, the effects of GF and circ_0059665 under hypoxic conditions on tumor growth were examined in vivo.

Compared with normoxic conditions, hypoxic conditions accelerated the tumor growth, while circ_0059665 silencing or GF treatment suppressed hypoxia‐induced tumor growth; moreover, hypoxic A549 cells containing sh‐circ_0059665 led to decreased tumor growth, with a more pronounced effect detected in the presence of sh‐circ_0059665 and GF (Figure S3a,b). The results of western blot and qRT‐PCR analyses suggested that the contents of circ_0059665 and NOVA2 were increased, while miR‐512‐5p expression was decreased in tumors under hypoxic conditions compared with normoxic conditions. Circ_0059665 knockdown or GF treatment reversed the increased circ_0059665 and NOVA2 level, as well as decreased miR‐512‐5p level in tumors under hypoxic conditions, and the effects of GF treatment on their expression changes were enhanced by circ_0059665 knockdown (Figure S3c–e). These results suggested that GF treatment suppressed NSCLC growth in vivo via circ_0059665 under hypoxic conditions.

The third and fourth paragraphs from the Discussion section should be corrected as follows:

Emerging evidence proposes that circRNAs can act as sponges for sequestering microRNAs (miRNAs) and then modulate the expression of downstream mRNAs.^29,30^ In a further mechanical analysis, we first identified the circ_0059665/miR‐512‐5p/NOVA2 axis in NSCLC cells. MiR‐512‐5p was observed to be decreased in NSCLC and was upregulated after GF treatment, indicating the antitumor potential of miR‐512‐5p in NSCLC, and previous studies have verified that miR‐512‐5p functions as a tumor suppressor in NSCLC.^31,32^ NOVA2 is a well‐recognized oncogene, and has been revealed to be elevated in cancer endothelial cells (ECs) of many types of cancers.^33^ In NSCLC, it has also been proven to promote NSCLC progression.^34–36^ In addition, hypoxia can induce NOVA2 expression in ECs, and is a potential NOVA2 regulator in tumor vasculature.^37^ In our study, we observed that hypoxia elevated NOVA2 expression in NSCLC cells, which was reduced by GF treatment. Moreover, forced expression attenuated the anticancer action of GF treatment or circ_0059665 knockdown.

In conclusion, GF alleviated NSCLC cell proliferation, invasion, and macrophage M2 polarization via the circ_0059665/miR‐512‐5p/NOVA2 axis in hypoxic conditions, indicating that GF in combination with circ_0059665 siRNA might be a promising therapeutic method for NSCLC.

Figures 4 and 7 caption should be corrected as follows:

FIGURE 4 Circ_0059665 targets miR‐512‐5p, and NOVA2 is a target of miR‐512‐5p. (a) Two predicted miRNAs (miR‐197‐3p and miR‐512‐5p) were identified in the overlapping of Circinteractome and CircBank databases. (b) Quantitative real‐time‐polymerase chain reaction (qRT‐PCR) analysis for miR‐197‐3p and miR‐512‐5p expression after circ_0059665 or pCD5‐ciR transfection. (c) The binding sites of miR‐512‐5p on circ_0059665. (d) The transfection efficiency of miR‐512‐5p or miR‐NC was validated by qRT‐PCR. (e, f) The interaction analysis between circ_0059665 and miR‐512‐5p by dual‐luciferase reporter assay. (g) qRT‐PCR analysis for miR‐512‐5p expression in non‐small cell lung cancer (NSCLC) and normal tissues. (h) Correlation analysis between circ_0059665 and miR‐512‐5p in NSCLC tissues. (i) qRT‐PCR analysis for miR‐512‐5p expression in NSCLC cell lines (H1299 and A549) and normal cell lines (16HBE). (j) qRT‐PCR for miR‐512‐5p level in H1299 and A549 cells after hypoxia stimulation alone or GF treatment and hypoxia stimulation. (k) The binding sites of miR‐512‐5p on NOVA2. (l, m) The interaction analysis between NOVA2 and miR‐512‐5p using dual‐luciferase reporter assay. (n) qRT‐PCR analysis for NOVA2 expression in NSCLC and normal tissues. (o) Correlation analysis between NOVA2 and miR‐512‐5p in NSCLC tissues. (p) Western blotting analysis for NOVA2 expression in NSCLC cell lines (H1299 and A549) and normal cell lines (16HBE). (q) Western blotting for NOVA2 level in H1299 and A549 cells after hypoxia stimulation alone or GF treatment and hypoxia stimulation. ***p < 0.001; ****p < 0.0001.

FIGURE 7 *Gentiana macrophylla* flavonoid (GF) treatment suppresses tumor growth of non‐small cell lung cancer (NSCLC) in vivo via circ_0059665. The in vivo growth curve (a), representative images, and the weight at the end points (b) of xenografts. (c, d) Quantitative real‐time‐polymerase chain reaction (qRT‐PCR) analysis for the levels of circ_0059665 and miR‐512‐5p in xenografts. (e) Western blotting analysis for NOVA2 protein level in xenografts. (f) Immunohistochemistry (IHC) analysis for the protein of NOVA2, c‐Myc, and MMP2 in xenografts. *p < 0.05.

References 31 and 32 should be corrected as follows:

31. Wang Z, Zhu X, Zhang T, Yao F. miR‐512‐5p suppresses the progression of non‐small cell lung cancer by targeting β‐catenin. *Oncol Lett*. 2020;19:415–23.

32. Cao B, Tan S, Tang H, Chen Y, Shu P. miR‐512‐5p suppresses proliferation, migration and invasion, and induces apoptosis in non‐small cell lung cancer cells by targeting ETS1. *Mol Med Rep*. 2019;19:3604–14.

We apologize for these errors.

